# Popliteal Artery Branching Variations: A Study on Multidetector CT Angiography

**DOI:** 10.1038/s41598-020-65045-6

**Published:** 2020-05-18

**Authors:** Serkan Oner, Zulal Oner

**Affiliations:** 1 Faculty of Medicine Department of Radiology, Karabuk University, Karabuk, Turkey; 2 Faculty of Medicine Department of Anatomy, Karabuk University, Karabuk, Turkey

**Keywords:** Anatomy, Interventional cardiology

## Abstract

Determining the branching pattern of the popliteal artery (PA) is an important step in planning some radiological and surgical procedures. The aim of this study was to investigate the course and morphology of the terminal branches of the popliteal artery using multidetector computed tomography (MDCT) angiography, and also to determine possible role gender in branching pattern. Three-hundred forty lower extremity MDCT angiography images for 170 patients (118 M, 52 F), who were between 20–80 years old, were examined. Popliteal artery branching types were grouped as percentage incidences. TPT diameters and lengths in Type IA extremities were compared based on gender and right or left side. Anterior tibial artery (ATA), posterior tibial artery (PTA) and peroneal artery dominance rates were calculated. 5000 times measurement data was mixed so that the cascade mean filter values were calculated for the right and left TPT length each time. It was observed that Type IA was the most common branching pattern (89.4%). The variational pattern incidence was 10.6% and the most common category was Type III (4.1%). The most common pattern was Type IB (3.2%). Variational pattern was 2 times more prevalent in females when compared to the males. The mean TPT diameter was 4.5 mm (2.7–7.3 mm) and there was no difference based on gender and the right-left side. The most common dominant artery for the right and left legs was PTA in both genders. The cut-off values calculated for the right and left TPT independent of gender were 31.30 ± 2.40 and 28.36 ± 2.58, respectively. Three new subtypes were identified as short (S ≤ 2 cm), standard (N = 2–4 cm) and long (L ≥ 4 cm) in Type IA, since it is in a wide variational range although it is a typical PA branching pattern.

## Introduction

The popliteal artery is a lower extremity artery that is a continuation of superficial femoral artery. After crossing the popliteal fossa, it branches into the anterior tibial artery (ATA) and tibioperoneal trunk (TPT) near the lower border of the popliteus muscle. TPT is then bifurcates into posterior tibial artery (PTA) and peroneal artery (PA) branches^1,2^. However, the popliteal artery branching pattern may exhibit variations based on embryological abnormalities. These variations were first indicated and classified in angiographic and postmortem studies in the literature^3^. The original classification system was first described in 1985 by Lippert and Pabst^4^ and transformed the most common system in 1989 by Kim *et al*.^5^. This classification system includes 10 subtypes in 3 primary types. The typing is based on the level of bifurcation. Popliteal artery exhibits bifurcation at the lower border of popliteus muscle in the classical anatomical description. However, since this cannot be determined angiographically, tibial plateau can be used as a reference point^2^.

Knowledge on popliteal artery and distal variations are important not only for the anatomists but also for interventional radiologists and surgeons. Anatomical variations in this region could play a significant role in the success of vascular grafting, surgical repair, transluminal angioplasty, embolectomy or knee operations with the increase in vascular surgeries and interventional procedures^6,7^.

Today, arterial variations are determined with multidetector computed tomography (MDCT) angiography, a non-invasive technique. CT angiography provides evaluation almost similar to normal anatomic structures with standard axial cross-sections, as well as three-dimensional reconstruction. This imaging technique is important in the patient follow-up, especially after a procedure, as well as the patient’s compatibility for endovascular or surgical treatment. Furthermore, the short application time benefits rapid evaluation of vascular structures for emergency surgery or endovascular interventions^8^.

The aim of the present study was to investigate the course and morphology of the terminal branches of the popliteal artery using MDCT angiography to minimize complications during interventional procedures, and also to determine if there was a difference between the genders.

## Results

In 170 evaluated patients, in both lower extremity popliteal artery branching pattern, a total of 15.3% variations were determined, including 144 normal patterns (84.7%), 16 unilateral variations (9.4%), and 10 bilateral variations (5.9%) (Table 1). Variational patterns were observed in 11.9% of the males and 23% of the females.

**Table 1:** Popliteal artery branching variation rates.

Pattern	Number of cases (n = 170)	Incidence (%)
Normal (Type IA)	144 (104 M, 40 F)	84.7
Unilateral variation	16 (10 M, 6 F)	9.4
Bilateral variation	10 (4 M, 6 F)	5.9

36 variational branching patterns were observed within a total of 340 lower extremities (10.6%). The incidence of variational patterns was as follows: Type IA: 89.4%; Type IB: 3.2%, Type IC: 1.5%, Type IIA1: 0.3%; Type IIA2: 0.6%; Type IIB: 0.9%, Type IIC: 0%; Type IIIA: 2%, Type IIIB: 1.5%; Type IIIC: 0.6% (Table 2).

**Table 2:** Popliteal artery branching patterns.

Branching type	Number of extremities	Incidence (%)	
Type IA	304	89.4	Type I 94.1
Type IB	11	3.2	
Type IC	5	1.5	
Type IIA 1	1	0.3	Type II 1.8
Type IIA 2	2	0.6	
Type IIB	3	0.9	
Type IIC	0	0	
Type IIIA	7	2	Type III 4.1
Type IIIB	5	1.5	
Type IIIC	2	0.6	

The shortest TPT was 9.1 mm, the longest TPT was 115.2 mm and both were observed in male patients. TPT size below 1 cm was observed in only two extremities. 69% of the TPT were between 28.31 and 31.65 mm. The mean TPT length in the lower extremities, which were evaluated as normal pattern (Type IA), were 31.30 ± 12.13 mm on the right side (p = 0.001) and 28.41 ± 13.34 mm on the left side (p = 0.000), and the mean TPT length was statistically higher on the right side (p = 0.002). The mean TPT diameters on the right and left sides were 4.63 ± 0.85 mm (p = 0.000), 4.56 ± 0.78 mm (p = 0.000), respectively. There was no significant difference between right and left TPT diameters (p = 0.256). Based on gender, the mean TPT length and diameter in males were 30.24 ± 13.07 mm (p = 0.000) and 4.60 ± 0.84 mm (p = 0.000), respectively and 28.82 ± 12.18 mm (p = 0.000) and 4.57 ± 0.76 mm in females (p = 0.000). There was no significant difference between male and female TPT length and diameters (p = 0.155 and p = 0.490, respectively) (Table 3).

**Table 3:** Tibioperoneal trunk (TPT) length and diameter.

TPT	Lenght	P value	Diameter	P value
Right	31.30 ± 12.13 29.85 mm (9.1–73.2 mm)	0.002*	4.63 ± 0.85 4.5 mm (2.7–7.3 mm)	0.256
Left	28.41 ± 13.34 26.05 mm (9.5–115.2 mm)		4.56 ± 0.78 4.5 mm (2.8–7.3 mm)	
Male	30.24 ± 13.07 28.40 mm (9.1–115.2 mm)	0.155	4.60 ± 0.84 4.5 mm (2.7–7.3 mm)	0.490
Female	28.82 ± 12.18 26.15 mm (10.7–96.3 mm)		4.57 ± 0.76 4.5 mm (3–7.3 mm)	

**Figure 1:**
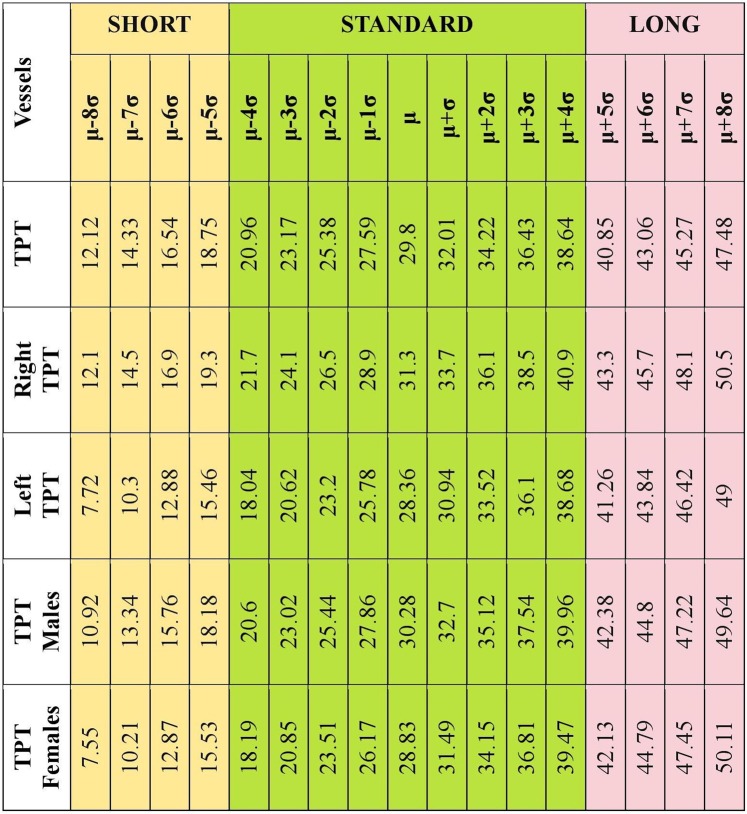
Distribution of all cut-off values around the arithmetic mean for TPT.

5000 times measurement data was mixed so that the cascade mean filter values were calculated for the right and left TPT each time. Thus, all possible cut-off values are calculated. The cut-off value for all TPT lengths was calculated as 29.8 ± 2.21. TPT cut-off values for the male and female patients were 30.28 ± 2.42 and 28.83 ± 2.66, respectively. The cut-off values calculated for the right and left TPT independent of gender were 31.30 ± 2.40 and 28.36 ± 2.58, respectively. Based on this data was used to classify the Type IA pattern in three subgroups as short (S ≤ 2 cm), standard (N = 2–4 cm) and long (L ≥ 4 cm) (Fig. 1).

In the evaluation of dominance among PTA, ATA and PA, there were 75 PTA (44%), 73 ATA (43%), 11 PA (6.5%) dominance and 11 ATA-PTA (6.5%) equal dominance in the right lower extremity. In the left lower extremity, 88 PTA (52%), 63 ATA (37%), 12 PA (7%) dominance and 7 ATA-PTA (4%) equal dominance were observed.

In the evaluation of dominance by gender, there were 56 PTA (54%), 31 ATA (30%), 13 PA (12%) dominance and 4 ATA-PTA (4%) equal dominance for both lower extremity in women. These rates were 107 PTA (45.5%), 105 ATA (44.5%), 10 PA (4%) dominance and 14 ATA-PTA (6%) equal dominance for men (Table 4).

**Table 4:** Dominance rates of poplitel artery terminal branches by direction and gender.

Dominant artery	PTA (%)	ATA (%)	PA (%)	ATA-PTA (%)
Right extremity (n = 170)	44	43	6.5	6.5
Left extremity (n = 170)	52	37	7	4
Woman (n = 104)	54	30	12	4
Man (n = 236)	45.5	44.5	4	6

## Discussion

Type IA was observed as the most common branching pattern (89.4%). The variational pattern incidence was 10.6% and the most common category was Type III (4.1%). The most common pattern was Type IB (3.2%). The variation rate for women were two times higher than in men. The most common dominant artery for the bilateral lower extremity was PTA in both gender. TPT length was in the range of 9.1–115.2 mm.

Popliteal artery branching variations are non-rare entity, occurring during the first 8 weeks of embryonic development as a result of mechanisms such as the persistence of primitive arteries, abnormal fusions of arterial structures and hypoplasia^9^. In the present MDCT angiography study, the incidence of variant branching patterns was 10.6% in all lower extremities. The angiographic studies conducted with relatively large patient groups in the literature reported incidences that varied between 6.5% and 18.7%^1,2,5,10,11^. An angiographic study conducted by Kil and Jung^12^ reported a similar finding with an incidence of 10.8%. Yanik *et al*.^13^ and Calisir *et al*.^14^ reported incidence rates of 16.4% and 13% in Turkish population using MDCT angiography, respectively, and determined a more frequent variant pattern when compared to the present study.

Kim *et al*.^5^ presented a new classification for popliteal artery branching pattern by modifying the Lippert system^4^, and indicated that this combined classification system would be better for surgeons due to clinical accessibility. Based on this classification, the Type I pattern was the most common branching pattern (94.6%) in the present study, consistent with several other studies^2,12,13^. Type IA, which is considered as the most common and normal pattern, was found in 89.4% of the cases in the present study, followed by Type IB with a 3.2% frequency. In several studies in the literature, Type IB was reported as the second most common branching pattern^5,11,14,15^.

The Kim *et al*.^5^ classification system, which is widely accepted in popliteal artery branching, is considered inadequate in the sub-classification of the Type IA pattern that constitutes the largest group. Because, the higher range of the TPT length is not taken into account in this classification, despite the fact that it plays an important role in the planning and selection of adequate material in endovascular treatment^10^. Certain reports emphasized the significance of this measurement in planning the infra-popliteal by-pass operation^5,16^. The cadaveric studies reported a mean TPT length between 30.3 ± 16.2 mm and 2–5 cm^17,18^. In the present study, TPT length was 30.24 ± 13.07 mm in males and it was 28.82 ± 12.18 mm in females with a wide range. Furthermore, a very long TPT (115.2 mm and 96.3 mm) in a case with two popliteal arteries branching in popliteus muscle inferior and 5–8 cm in 11 lower extremities. Previously, Demirtas *et al*.^15^ reported a very long TPT (110 mm) and proposed a new subtype classification (Type D). Celtikci *et al*.^10^ reported the mean TPT length as 30.5 mm (7.7–137.2 mm) in extremities with Type IA pattern and mentioned the requirement for a new sub-classification in this group and proposed to classify Type IA as Type IA S (short) and Type IA L (long) with a cut-off value of 3 cm. The present study findings, which coincide with the literature, show that TPT length is mostly between 28,31 and 31,65 mm (69%). Considering all these results Type IA pattern could be divided into three subgroups as Type IA short (S ≤ 2 cm), standard (N = 2–4 cm) and long (L ≥ 4 cm) based on the TPT length. Different from the studies in the literature, there was no difference between the TPT diameter values based on gender.

The frequency of popliteal artery with high branching (Type II) ranged between 1.6% and 7.8% in previous studies^1,2,5,11–15^. In the present study, this frequency was 1.8% and it is located in low results in the literature. The most frequently observed pattern was Type IIB (0.9%) in this group, consistent with previous literature. We did not observe any Type IIC pattern, consistent with the previous studies^12–15^ in the literature. This variation was reported lower than 0.2% in certain other studies^2,5,11^. Furthermore, Mavili *et al*.^11^ described the Type IID pattern (trifurcation on tibial plateau) as a new pattern.

The frequency of Type III branching pattern was reported to be between 1% and 7.6%^2,5,11,12^. In the present study, it was determined that this frequency was 4.1%, which was consistent with other MDCT angiography studies^13–15^. In this group, Type IIIC pattern was the rarest variation (0.1–0.8%) and only one case was identified in MDCT angiography studies^15^. We identified Type IIIC pattern in two lower extremity (0.6%).

In other studies conducted with MDCT angiography, Yanik *et al*.^13^ reported no Type IIB, IIC or IIIC patterns and Calisir *et al*.^14^ reported no Type IIC and IIIC patterns. Demirtas *et al*.^15^ reported that they did not observe any Type IIC pattern similar to the present study.

In the present study, it was determined popliteal artery branching variations were about twice as frequent in females when compared to males (23%, 11.9%). In the literature, gender-based comparisons are limited and no significant differences between the variation rates in females and males were reported in previous studies^10^. Furthermore, we assessed the dominance prevalence differences between the popliteal artery terminal branches. In the present study, the most frequent dominant artery on both the right and left side was PTA for both genders.

Knowledge on popliteal artery branching variations is important in clinical procedures conducted by vascular surgeons and interventional radiologists. It can also help radiologists to avoid misinterpretation of imaging findings during radiological examinations^11^. The knowledge on Type II patterns with high bifurcation between the popliteus muscle and the posterior tibial cortex is important for orthopedic interventions such as total knee arthroplasties, high tibial osteotomies, posterior cruciate ligament reconstructions, lateral meniscal repairs and arthroscopies^19^. In the presence of Type III variation, it may be necessary to change the technique of extremity angioplasty^20^. It may also be necessary to review the procedure in patients with a Type III pattern who are scheduled for a lower extremity fibular free flap. Because the peroneal artery, which is the only artery that supplies the foot, could be damaged, leading to ischemia^21^.

MDCT angiography, has several advantages such as short application time, thin sections, three-dimensional images, high spatial resolution, the ability to detect vascular pathologies and extraluminal pathologies with MDCT technology^22^. Compared with DSA, it was reported that MDCT angiography has a sensitivity and specificity between 93% and 100% in evaluating peripheral arterial disease^23^. The results of this study, which we performed using MDCT angiography, are more accurate than the cadaveric studies in the literature. Because vascular structure shows postmortem changes. In the literature, there are two studies that analyzed the popliteal artery branching pattern on 126 and 742 extremities using 64-section MDCT on the lower extremity^13,14^ and one study that analyzed on 1261 extremities using 128-section MDCT^15^. We analyzed 340 lower extremities using 16-section MDCT in the present monocenter study. MDCT angiography findings were not correlated with clinical findings. These can be considered as relative limitations in the present study. Furthermore, 85 patients were excluded due to factors such as arterial stenosis-occlusion and surgery history and unavailability of the contrast agent. This exclusion may have affected the findings. Differences between the findings in other studies in the literature may be related to the following factors: number of cases analyzed, study methodology (cadaveric or angiographic) and regional/racial differences.

In conclusion, this study revealed that there was a 10.6% variation in popliteal artery branching and twice as frequent in women. The most common variational pattern is Type IB. Since the TPT length is in a wide range, we identified new three subtypes (Type IA S, N, L) in Type IA considered usual pattern. PTA is the most dominant of the popliteal artery terminal branches in both genders.

## Methods

### Study population

The lower extremity CT angiography images of 170 patients (118 M, 52 F), who were admitted with various indications between January 2015 and December 2018, were used. The mean age of male patients was 57 and the mean age of female patients was 58. Patients without severe atherosclerotic vascular disease, arterial stenosis/occlusion, and history of femoropopliteal by-pass operation were included in the study.

### Multidetector CT protocol

All images were obtained with a 16-slice MDCT system (Aquilion 16; Toshiba Medical Systems, Tokyo, Japan). The patients were placed in the supine position with their feet first in the gantry. All lower extremities were included in all cases and image parameters were as follows: section thickness: 1 mm, tube voltage: 100–120 kV, gantry rotation: 0.75 s, and pitch value: 1.0 mm. The contrast agent bolus was applied with an automatic injector pump. The contrast agent Omnipaque 350 ® (Iohexol, GE Healthcare, Milwaukee, WI, USA) that was used in all applications was between 110 and 150 ml, depending on the body mass of the patient. The contrast agent administration rate was 3.5 ml/sec.

### Image analysis

All CT images were evaluated by the consensus between an experienced radiologist and an anatomist on a workstation (Vitrea v6.1, Vital Images, Plymouth, MN, USA). Axial plan images were analyzed in the vascular window with two- and three-dimensional reconstructions (Maximum Intensity Projection-MIP, Volume Rendering) and popliteal artery branching patterns were noted. In patients with Type IA pattern, the images were adjusted to the same magnification on the coronal plane and to measure the length and diameter. The dominance was assessed based on ATA, PTA and PA diameters.

### Classification of TPT

**Figure 2:**
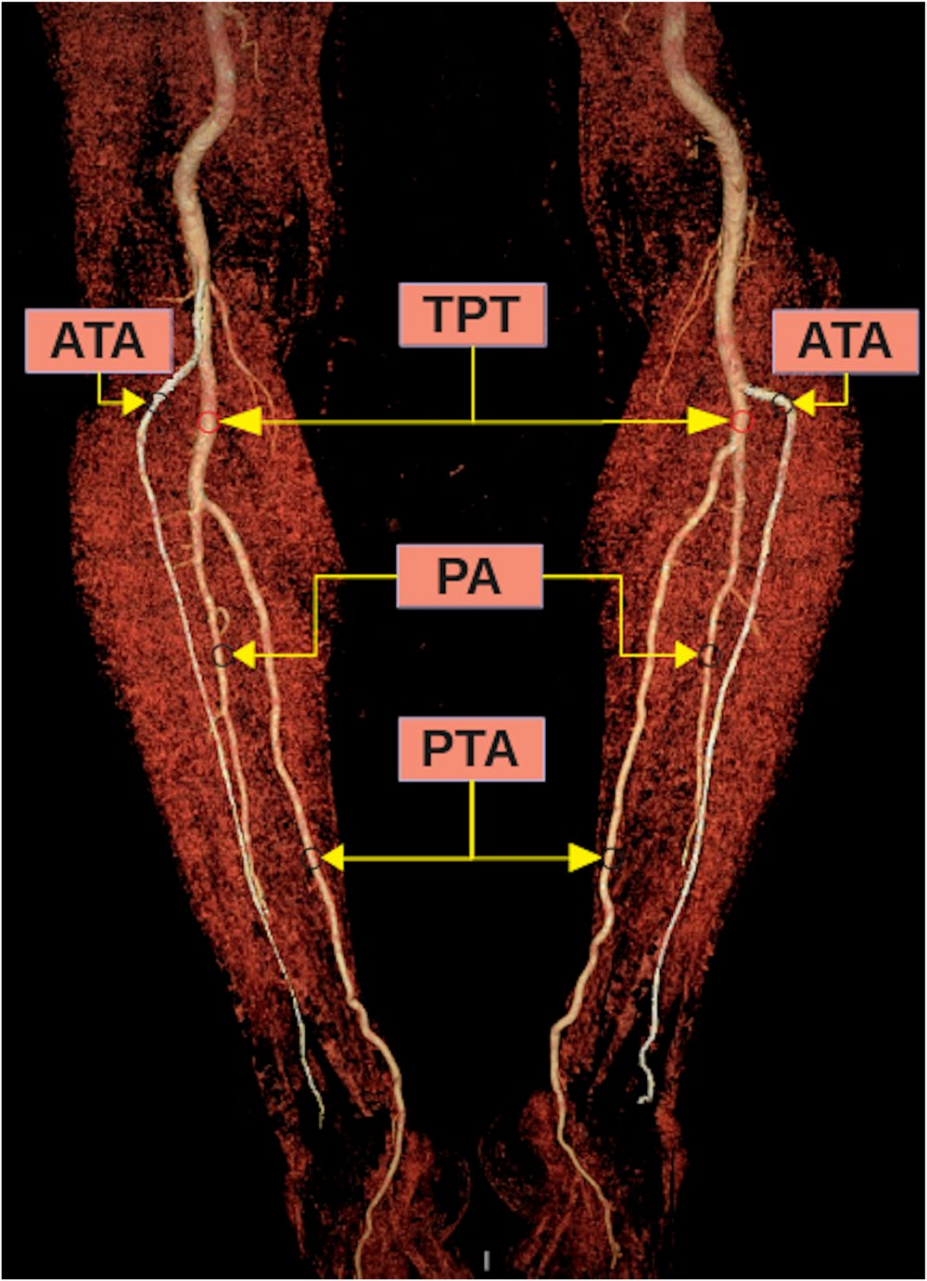
Three-dimensional volume rendered image shows the Type IA pattern on the left limb of the same patient and the Type IIA1 pattern on the right limb. TPT: Tibioperoneal trunk, ATA: Anterior tibial artery, PA: Peroneal artery, PTA: Posterior tibial artery.

**Figure 3:**
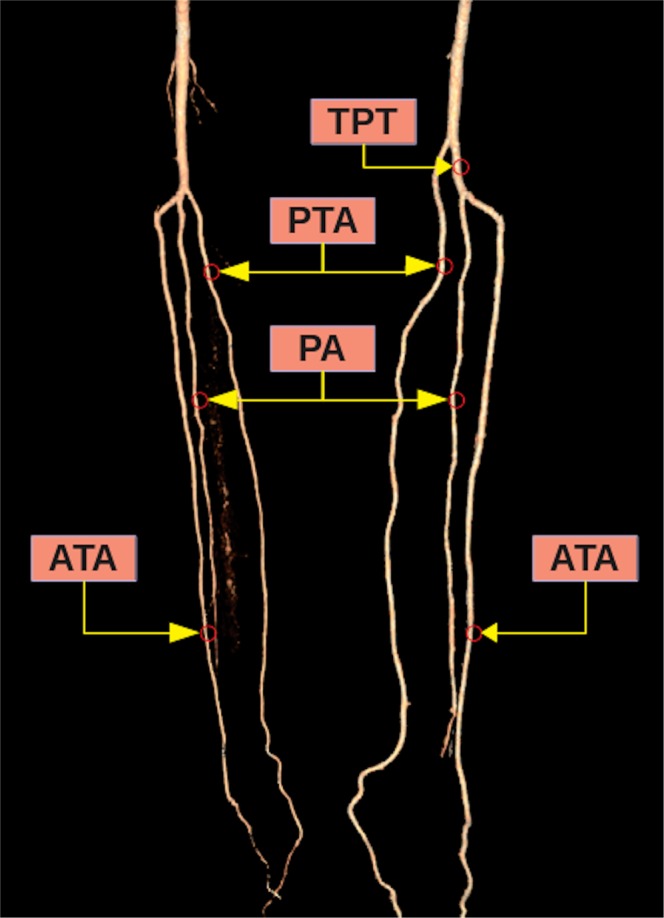
Three-dimensional volume rendered image shows the Type IC pattern on the left limb of the same patient and the Type IB pattern on the right limb. TPT: Tibioperoneal trunk, PTA: Posterior tibial artery, PA: Peroneal artery, ATA: Anterior tibial artery.

**Figure 4:**
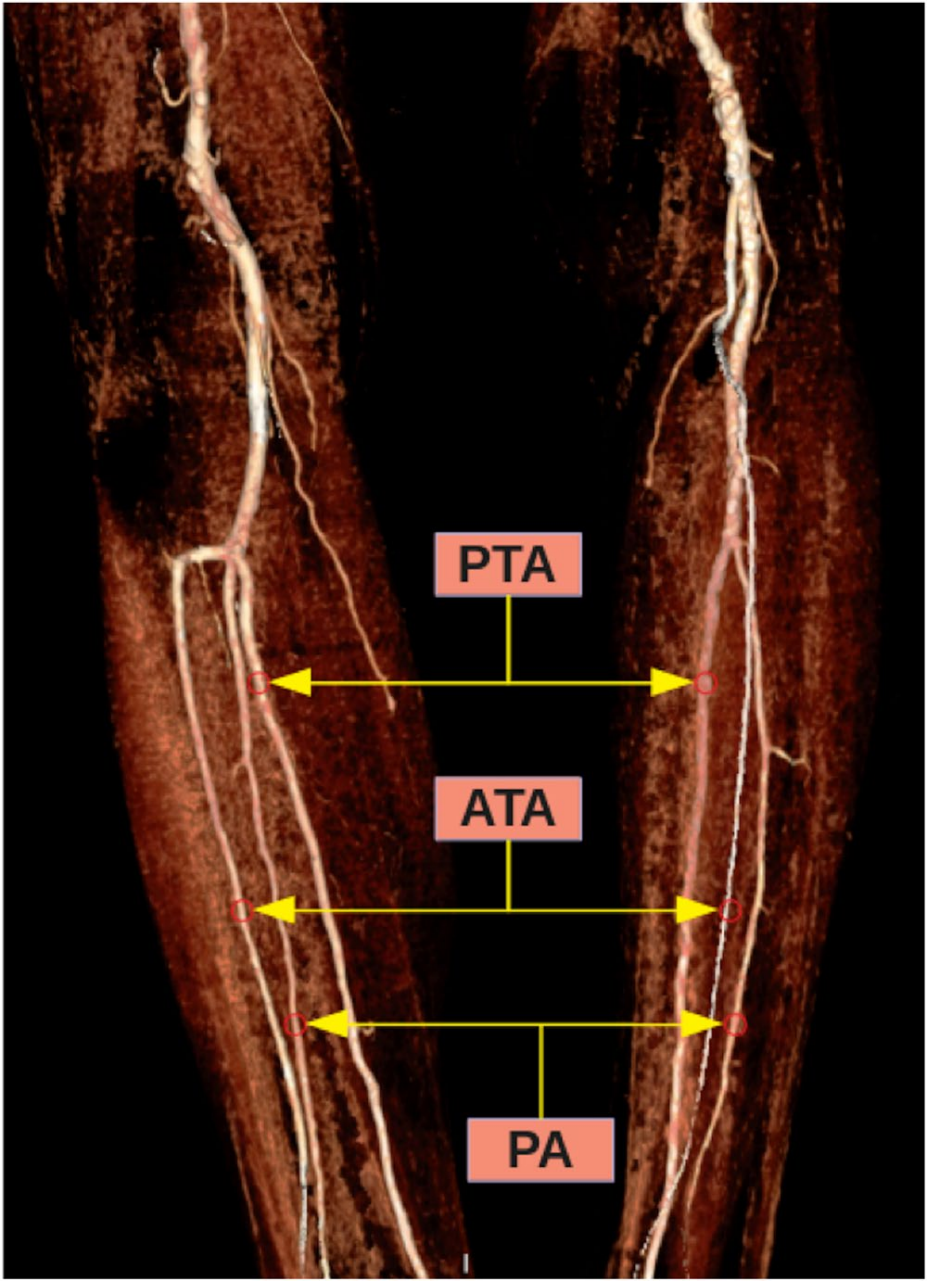
Three-dimensional volume rendered image shows the Type IIA2 pattern on the left limb of the same patient and the Type IB pattern on the right limb. PTA: Posterior tibial artery, ATA: Anterior tibial artery, PA: Peroneal artery.

**Figure 5:**
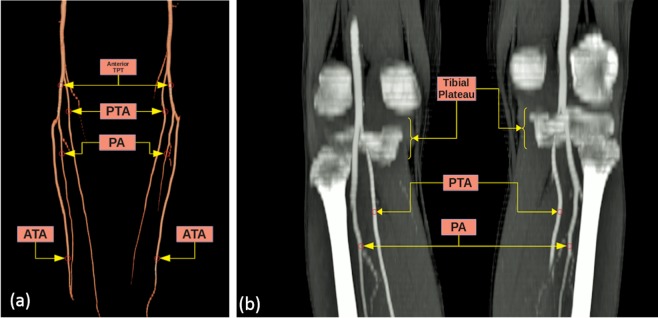
Three-dimensional volume rendered image (**a**) and the CT angiography image (**b**) show the Type IIB pattern on both limbs of the same patient. TPT: Tibioperoneal trunk, PTA: Posterior tibial artery, PA: Peroneal artery, ATA: Anterior tibial artery.

**Figure 6:**
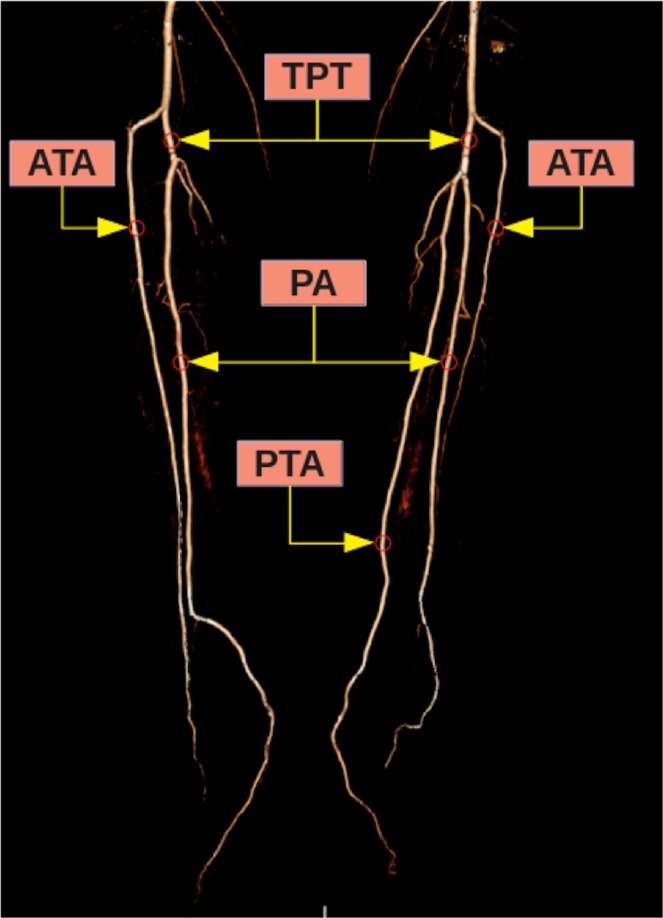
Three-dimensional volume rendered image shows the Type IIIB pattern on the left limb of the same patient and the Type IIIA pattern on the right limb. TPT: Tibioperoneal trunk, ATA: Anterior tibial artery, PA: Peroneal artery, PTA: Posterior tibial artery.

**Figure 7:**
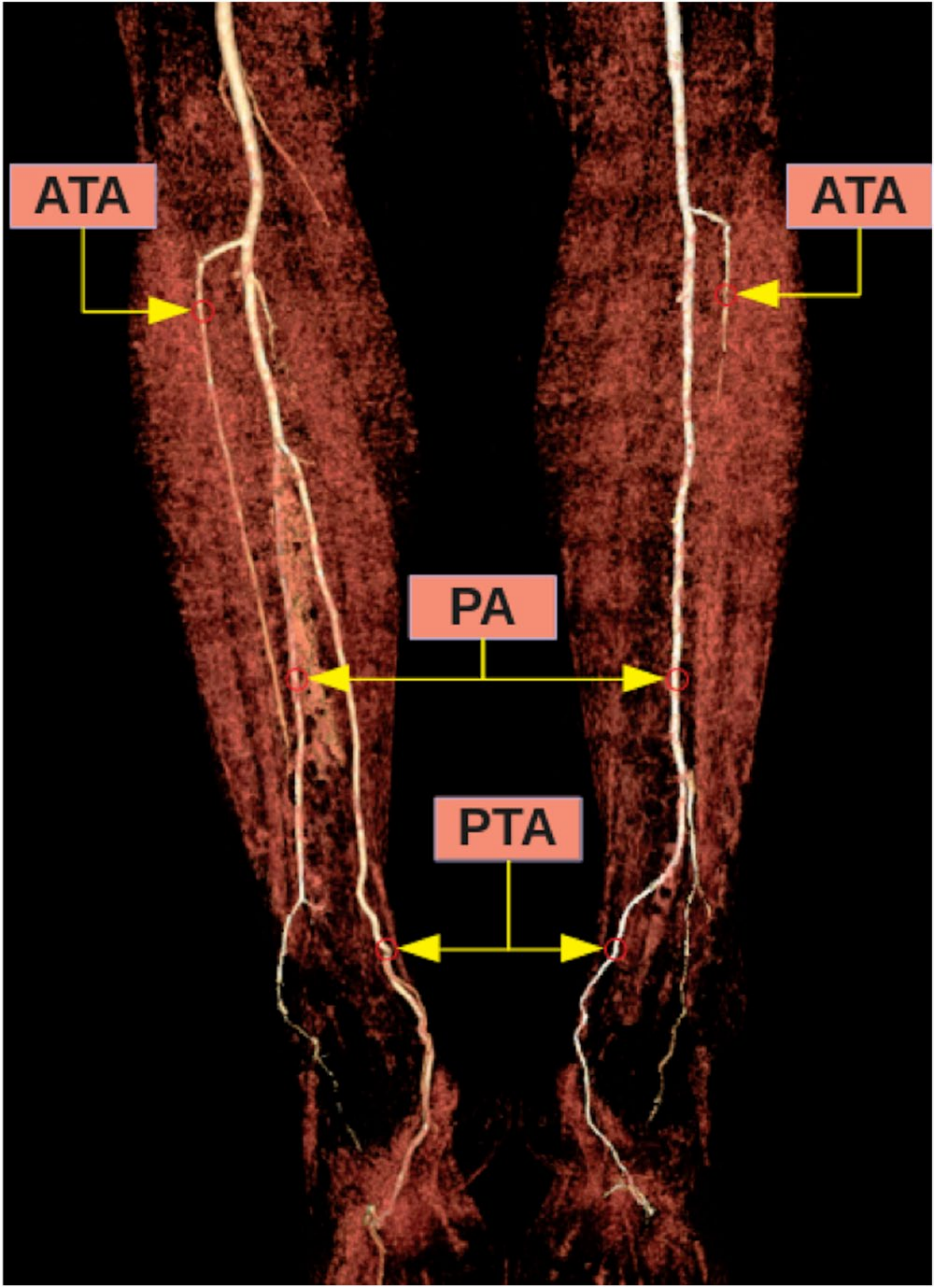
Three-dimensional volume rendered image shows the Type IIIB pattern on the left limb of the same patient and the Type IIIC pattern on the right limb. ATA: Anterior tibial artery, PA: Peroneal artery, PTA: Posterior tibial artery.

According to the classification reported by Kim *et al*.^5^, popliteal artery branching was categorized in 10 groups: the normal level branching under the tibial plateau (the popliteus muscle lower boundary) was classified as Type I. Type IA (common type): ATA is the first branch and continues as posterior TPT and is divided into PTA and PA bifurcations (Fig. 2). Type IB (trifurcation type): ATA, PTA and PA are branched within 5 mm of each other without the TPT (Figs. 3 and 4). Type IC: PTA is the first branch, and then continues into the anterior TPT and is divided into ATA and PA (Fig. 3). Type IIA1: ATA is branched on the tibial plateau and then follows the normal course (Fig. 2). Type IIA2: ATA branches on the tibial plateau, first bends medially and then resumes in the normal location (Fig. 4). Type IIB: PTA branches as the first branch on the tibial plateau and then continues as the anterior TPT and bifurcates as ATA and PA (Fig. 5). Type IIC: PA branches as the first branch on the tibial plateau and then divides into the ATA and PTA from the common trunk. Type IIIA: PTA is hypoplasic and is supported by the PA distally (Fig. 6). Type IIIB: ATA is hypoplasic and supported by the PA distally (Figs. 6 and 7). Type IIIC: ATA and PTA are hypoplasic and supported by the PA distally (Fig. 7).

### Determination of cut off value

**Figure 8:**
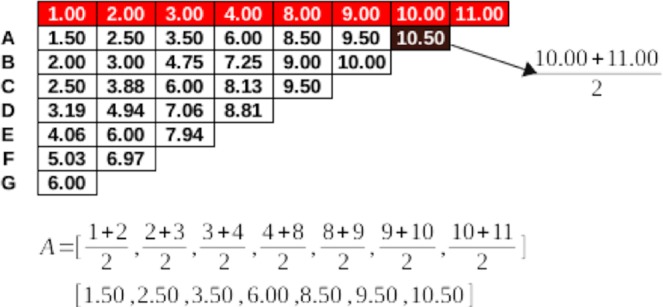
The calculation of cascade mean filter value.

Randomized cascade mean filter method was used to distinguish short and long sizes in measured TPT length values. In order to calculate the cut-off value, arithmetic mean of the measurements was calculated until a single value was left in the cascade form^24,25^. The numbers in the desired array to obtain cascade mean filter value was consecutively subjected to the arithmetic mean processing. Each calculated average was stored in a different array. Subsequently, successive arithmetic averages were taken from the averages obtained and retained in an array. In this way, an array of length was reduced in each iteration. The process was repeated until the last single value remains. The last value obtained was called cascade mean filter value and used as a cut-off value to apply a threshold to the initial array. The calculation of the cascade mean filter value was schematically presented in Fig. 8. Here, by the nature of the method, the cascade mean filter value will change when the locations of the numbers in the first array change. To eliminate this effect, the cascade mean filter value was calculated by mixing the first array each time. In this study, the number of repetitions was determined as 5000. After 5000 repetitions, the mean and standard deviation of all values obtained was used to divide the first array up to the desired class. In this study, three main classes were considered and the classes were defined as short, normal and long. Dividing operations were carried out from normal to long by adding standard deviation to the arithmetic mean value, and from normal to short as subtracting standard deviation from the arithmetic mean value. The number of repetitions of this process was determined according to the case where the minimum number was lowered in the first array.

### Statistical analysis

Minitab ® statistics software (version 17.3.1, State College, PA) was used in statistical analysis. The normal distribution of the data was determined with Shapiro-Wilk normality test. Mann Whitney U test was used to compare the data. p < 0.05 was considered as statistically significant. Data were presented as mean ± SD and median (min-max).

### Ethical considerations

This retrospective study was approved by Local Non-Interventional Clinical Trials Ethics Committee with the protocol number 14/36.

### Ethical approval

The present study was approved by Local Non-Interventional Clinical Trials Ethics Committee with the protocol number 14/36. All procedures performed in studies involving human participants were in accordance with the ethical standards of the institutional and/or national research committee and with the 1964 Helsinki declaration and its later amendments or comparable ethical standards.

### Informed consent

This study is retrospective and based on images taken from the hospital archive system. Therefore, no informed consent is required and ethics committee approval was obtained accordingly.
